# Seroprevalence of hemorrhagic septicemia in dairy cows in Assam, India

**DOI:** 10.1080/20008686.2019.1604064

**Published:** 2019-05-01

**Authors:** Rajeswari Shome, Ram Pratim Deka, Swati Sahay, Delia Grace, Johanna F. Lindahl

**Affiliations:** a ICAR- National Institute of Veterinary Epidemiology and Disease Informatics (ICAR-NIVEDI), Bengaluru, India; b Department of Biosciences, International Livestock Research Institute (ILRI), Guwahati, India; c Department of Clinical Sciences, Swedish University of Agricultural Sciences, Uppsala, Sweden; d Department of Biosciences, International Livestock Research Institute (ILRI), Nairobi, Kenya; e Department of Biosciences, International Livestock Research Institute, Hanoi, Kenya; f Department of Medical Biochemistry and Microbiology, Uppsala University, Uppsala, Sweden

**Keywords:** Pasteurella multocida, pasteurellosis, South Asia, risk factors, dairy production, serology

## Abstract

Hemorrhagic septicemia (HS) is a highly fatal disease caused by *Pasteurella multocida* that often cause outbreaks in buffalo and cattle in India, and thus is a major cause of production losses. It is one of the livestock diseases with the highest mortality, and despite available vaccines, outbreaks still occur. To assess the seroprevalence in the state of Assam, Northeast India, 346 serum samples from cows from 224 randomly selected households, from both urban and rural areas of three districts, were tested with a commercial ELISA. In total 88 cows were seropositive (25.4%), and indigenous cattle were significantly more seropositive (33.5%) compared to the crossbred cattle (18.5%) (p = 0.002). Herd prevalence was 35.7%, and more rural farms (47.4%) were positive compared to the urban farms (23.6%) (p < 0.001). No other risk factors were identified in this study. Only one farm had vaccinated against HS, but there were no seropositive animals detected in that herd. This study shows that HS is highly prevalent in Assam. Considering the importance of dairy production in India, and the dependence of the rural Assam population on farming and livestock keeping, more extensive vaccination campaigns would be important.

## Introduction

Hemorrhagic septicemia (HS) is a highly fatal, acute septicemic disease in buffalo and cattle, caused by mainly two specific serotypes of *Pasteurella multocida* designated B:2 (Asian serotype) and E:2 (African serotype), but other serotypes have been shown to circulate in India [1–4]. Outbreaks of HS may have case fatality rates above 80% [5]. In India, HS was responsible for the highest mortality of infectious diseases in buffaloes and cattle during 1974–1986 [6], and has been estimated to cause economic losses of USD 792 million per year for the livestock industry [7].

Although several vaccine formulations are commercially available, outbreaks still occur every year in India. Vaccination campaigns at the onset of the outbreak are not efficient to stop high mortalities, and hence, identifying HS endemic areas to prioritize compulsory vaccination in the state is important to prevent outbreaks. The HS outbreak trends remain similar for the last 5 years in the state of Assam, North-East India [8]. This study was undertaken to record the seroprevalence and identify risk factors in Assam.

## Materials and methods

The study was approved by Institutional Animal Ethics Committee of ICAR-National Institute of Veterinary Epdemiology and Disease Informatics (ICAR-NIVEDI), all farm owners were informed about the study and gave consent to participate and to publish the data.

This study was conducted in Assam, a state of northeastern part of India through a cross-sectional survey from September to December 2016. Assam has a temperate climate (summer at 35–38°C and winter at as low as 6–8°C in some parts of the state) and heavy rainfall and high humidity prevailing in the state. In the first stage, three sample districts were purposefully selected which included two representative districts (Baska and Golaghat) along with the capital district (Kamrup) of the state. Availability of primary laboratory support and safety of the study team were considered during the selection of the districts. Two community development blocks (CDBs) (one rural and one urban) from each district were randomly selected. From each of the six CDB, four villages were selected randomly. From each selected village, 10 dairy-producing households were selected randomly and from each selected household, maximum three mature female animals were selected randomly for the collection of a blood sample for serological survey. Random selection was done by computer-generated random numbers. The required sample size was calculated to be at least 196 (estimated prevalence of 15%, 95% level of confidence and 5% precision) [9], and in total 224 households were interviewed with a pre-tested questionnaire on farming system and basic farmers details. From the interviewed households, 346 serum samples were collected with the history of animals. No buffalo was found in the interviewed households.

All the sera samples were tested in singles for anti-*P. multocida* antibodies using pasteurellosis enzyme-linked immunosorbent assay (ELISA) (LT Biotech, Vilnus, Lithuania) antibody Kit as per the manufacturer’s instructions and the results were interpreted based on the conversion of the optical densities to percent positivity (PP) values, with values ≥32 deemed positive. The manufacturer provided no data on specificity and sensitivity, thus all presented data are apparent prevalence.

Data were entered in excel and analyzed using Stata 14 (STATACorp Ltd) and descriptive statistics of proportions, mean, variance and standard deviation (SD) were conducted. Univariable associations between binary or categorical variables were studied by χ^2^ test.

## Results

Out of the 224 farms, 80 had at least one positive animal (35.7% herd prevalence, 95% confidence interval (CI) 29.4–42.4), four had two positive animals, and in two farms all three tested animals were positive. Significantly fewer (p < 0.001) urban farms (23.6%) were positive compared to rural (47.4%) (Table 1).

**Table 1.** Risk factors on farm and cow level for seropositivity for *P multocida* in Assam, India.

	Seropositive	Chi 2	P-value	Odds ratio (95% CI)
**Herd level risk factors**				
Location				
Urban	26/110 (23.6%)			
Rural	54/114 (47.4%)	13.7	P < 0.001	2.9 (1.6–5.2)
Districts				
Kamrup	26/75 (34.7%)			
Baska	28/75(37.3%)			
Golaghat	26/74 (35.1%	0.13	p > 0.9	
Cow cleanliness level				
Very clean	29/74 (39.2%)			
Average	49/147 (33.3%)			
Dirty	1/2 (50.0%)	2.73	P = 0.4	
Cow shed cleanliness level				
Very clean	29/69 (42.0%)			
Average	44/139 (31.7%)			
Dirty	6/15 (40%)	4.12	P = 0.25	
Floor type				
Concrete	70/191 (36.7%)			
Not concrete	9/32 (28.1%)	2.68	P = 0.26	
Disinfection frequency				
Daily	1/2 (50%)			
At least 1/week	4/16 (25.0%)			
At least every month	14/46 (30.4%)			
More seldom	7/22 (31.8%)	2.38	P = 0.67	
Veterinary consultation frequency				
Never	21/51 (41.2%)			
Weekly (more than once per week)	0/3 (0%)			
Monthly (more than once per month)	7/24 (29.2%)			
Yearly	28/83 (33.7%)			
More seldom	24/63 (38.1%)	3.07	P = 0.55	
Training on farm management				
Farmer had no training	73/202 (36.1%)			
Farmer had training	7/22 (31.8%)	0.16	P = 0.69	
Training on diseases				
Farmer had no training	77/211 (36.5%)			
Farmer had training	3/13 (23.1%)	0.96	P = 0.33	
Rearing system				
Fully stall fed/tied stall,	19/64 (29.7%)			
Partly stall fed (grazing part time)	61/160 (38.1%)	1.42	P = 0.23	
Introduction of new animals				
Did not introduce animals last year	65/191 (34.0%)			
Did introduce animals last year	15/33 (45.5%)	1.60	P = 0.21	
Quarantine routines				
No quarantine	79/219 (36.1%)			
Has quarantine	1/4 (25.0%)	0.77	P = 0.7	
**Cow level risk factor**				
Breed				
Cross-breed	31/168 (18.5%)			
Local breeds	55/164 (33.5%)	9.8	0.002	2.2 (1.3–3.7)

In total, 88 animals were positive (25.4%, 95% confidence interval 20.9–32.4), with higher seroprevalence recorded in the indigenous cattle (33.5%) compared to crossbreed (18.5%) (p < 0.002). The highest seroprevalence was in the age group of 5–10 years (27.4%) (Table 1).

Out of the 65 (29%) households that reported that they had vaccinated their cows, 56 knew which disease they had vaccinated with one farm reportedly vaccinated against HS, and the other farm's foot and mouth disease only. In the HS vaccinated farm, all tested animals were seronegative.

## Discussion

This study found an HS seroprevalence of 35.7% in three districts of Assam. Even though the three selected districts were geographically distantly located, no significant differences in the herd prevalence were observed. The farms in rural household showed significantly higher sero-prevalence compared to the urban farms. Previous findings that HS is more common in large and free-roaming herds than in smaller, well-managed and stall-fed herds [10] would support this, but our study could not find significant associations with the management system.

This study showed indigenous breeds having higher sero-positivity (33.5%) compared to crossbred animals (18.5%), which can be explained by these mainly being kept in rural areas. It has been reported that younger animals are more susceptible than older to HS [2], which is consistent with our study that had lower prevalence among the oldest age category.

Dairy production is very important in Assam, and 82% of the households have been reported to keep cattle, most commonly in small herds of up to eight animals [11]. The high sero-positivity observed may be due to factors such as rice paddy crop cultivation, which in Assam accounts for more than 90% of the total food crop area [12], which may favor outbreaks [2]. There was only one farm that had vaccinated against HS. Interestingly, in the HS vaccinated farm, all tested animals were sero-negative. Vaccination is important for prevention of HS, and in HS outbreaks disease has been observed more commonly in unvaccinated animals [13,14]. Since dairy production is very important for the rural population in Assam, vaccination against HS is an important way towards reducing losses.
